# Comparison of Different Liquid and Semisolid Vehicles Selected for Oral Administration of Pellets and Minitablets with Diazepam: *In Vitro* Investigation

**DOI:** 10.1208/s12249-020-01761-6

**Published:** 2020-07-31

**Authors:** Hanna Kotlowska, Marta Szymanska, Malgorzata Sznitowska

**Affiliations:** 1 Department of Pharmaceutical Technology, Medical University of Gdansk, Al. Hallera 107, 80-416 Gdansk, Poland; 2 Student Chapter of the ISPE, Department of Pharmaceutical Technology, Medical University of Gdansk, Gdańsk, Poland

**Keywords:** pellets, minitablets, sprinkle, disintegration, dissolution

## Abstract

The acceptability and palatability of a dosage form are extremely important to improve patient compliance. Mixing oral solid dosage forms with food carriers is often necessary to ease swallowing and provide the taste-masking effect. The present research investigated how a liquid or semisolid carrier influences the disintegration time and drug dissolution rate of pellets and minitablets with diazepam. The disintegration of pellets and minitablets in liquid carriers (water, milk and apple juice) was determined using a texture analyser. Dissolution tests were performed for the dosage forms dispersed in gel vehicles (2% carmellose and 0.5% carbomer gels) or applesauce. The disintegration of minitablets in water and apple juice was fast (1 min), but it slowed to 3 and 5 min in milk and gel vehicles, respectively. The pellets disintegrated in liquid carriers within 3 min. The drug dissolution rate in 0.1 M HCl depended on the gel viscosity in this medium. The preserved high viscosity of a carmellose gel inhibited the dissolution of diazepam. On the other hand, the viscosity of the carbomer gel decreased rapidly, and in effect, the dissolution rate of diazepam from the incorporated pellets or minitablets was comparable to the dissolution from loose pellets or minitablets.

**Figure:**
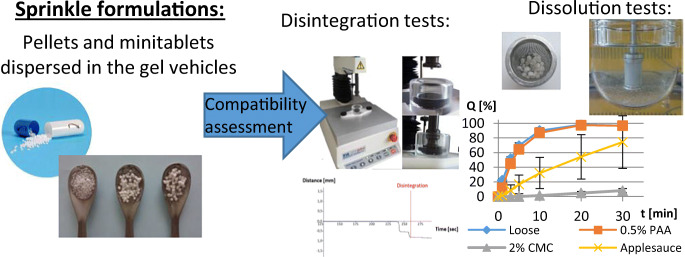
Graphical abstract

Graphical abstract

## INTRODUCTION

Swallowing difficulties (dysphagia) are becoming more common. Approximately 70–90% of the elderly and 25–45% of children experience swallowing problems (1). The lack of commercially available, age-appropriate medicinal products forces patients and caregivers to manipulate the dosage form, including breaking or crushing the tablets or mixing them with food or drink. All of these actions may reduce clinical efficacy or increase the risk of adverse reactions when the chemical and physical stability or bioavailability is affected (2). Sprinkles are a new medicinal product of multiparticulate solid oral dosage forms dedicated for mixing with soft food or liquids (3).

Sprinkles occur as beads (e.g., granules, pellets, or minitablets) packed in capsules or sachets that are designed to be removed and mixed with a liquid or a soft food before administration, to improve swallowability. The carrier supports the taste-masking properties, which significantly improve patient compliance. Several studies showed that sprinkles are more acceptable by children and their caregivers than liquid dosage forms (4–6). Sprinkle formulations are gaining more popularity because they are presented as single-dose, preservative-free and taste-masked products (7).

The beads may be coated to mask the unpleasant taste of the active substance (8). The coating of multiparticulate formulations also provides the opportunity to modify the release kinetics of the active substance. There are enteric and sustained release products in which polymers are soluble in pH above 5.5 or insoluble but permeable for water, respectively. The manufacturing of modified-release dosage forms that are acceptable for children is especially important due to the small number of such products available on the market (9).

According to the FDA (Food and Drug Administration) guideline from 2012, the maximum size of beads formulated as sprinkles is up to 2.8 mm, which is the size that does not stimulate the urge to chew (10). It is especially important in cases of the modified-release multiparticulates, where unintended chewing may change the pharmacokinetics of the active pharmaceutical ingredient (API) (11). The pilot clinical study of Kluk et al. (12) demonstrated that preschool children easily swallowed several minitablets (2 and 3 mm) when they were sprinkled on a fruity jelly. However, the authors emphasized the relatively high frequency of chewing (even 23%) because children from an early age are taught to bite or chew food to eliminate the risk of choking.

The recently published FDA draft guidance (13) proposes *in vitro* methods for testing the sprinkle drug product and the vehicle mixture. It is important to standardize a methodology that supports the selection of an appropriate vehicle. The vehicle choice was often based on the acceptable taste and texture rather than on physiochemical characteristics, which may affect the oral bioavailability or stability of the API (14). The need to demonstrate that the drug product quality is maintained when it is mixed with the vehicle is highlighted in the guideline. A 2-h stability test of drug product-vehicle mixture is recommended, and some general recommendations for the dissolution testing are also provided. The drug release profile from the original product and the product-vehicle mixture as determined with pharmacopoeial tests should be consistent. Any significant differences *in vitro* may indicate a risk of change in bioavailability; however, the *in vitro* testing cannot replace the *in vivo* food-effect studies, especially for modified-release products (15).

The most commonly used liquid carriers for sprinkling are apple juice or breast and infant formula milk, and common soft foods are yogurt, pudding, fruit and vegetable-based baby food, especially applesauce. Due to the natural origin of these products, any changes in the composition due to regional or climate conditions cannot be excluded. Therefore, standard suspending vehicles appeared on the market. An example is Ora^®^-Sweet, which is a suspending, flavouring and sweetening liquid vehicle. However, it is primarily used in pharmaceutical compounding as a vehicle in oral suspensions (16). A commercially available lubricating gel with carrageen and maltodextrin (Gloup Original) is used to facilitate the swallowing of solid oral drugs in children from 2 years of age. It is described as safe with no effect on the dissolution rate of paracetamol from tablets (17). Kluk and Sznitowska (18) defined the physical properties of a semisolid gel that is appropriate for the administration of minitablets and pellets. Viscosity, texture/ductility, spilling risk from a spoon under shaking and the disintegration time of minitablets in the gels were examined. The tests were designed to imitate the real conditions that occur during the administration of a medicine. The best application properties were demonstrated for medium viscosity gels, such as 1% or 1.5% carmellose sodium gels and 0.25% or 0.5% carbomer gels.

In the present study, immediate release pellets and minitablets with diazepam were formulated. Diazepam is a benzodiazepine derivative anxiolytic drug. It is a lipophilic compound (logP = 2.8) with limited solubility in water, less than 50 μg/ml (19). Diazepam is more often prescribed for elderly patients, and it is available on the market as tablets in strengths of 2 mg and 5 mg. The drug is also used in children for premedication, but the dosage accuracy of 0.3 mg/kg body weight is required in this case (20). The oral solution is available for children from 6 months; however, it contains alcohol as co-solvent and the stability after the first opening is limited (21). Solid oral multiparticulate dosage forms (pellets and minitablets) exhibit better stability and allow flexible dose adjustments in children of different ages. The study tested the *in vitro* disintegration in liquid and semisolid carriers, and the dissolution rates for the dosage forms dispersed in the hydrophilic gels and applesauce were determined. The study examined any possible influence of the carrier on the performance of solid dosage forms in *in vitro* conditions and justified the methodology of these tests in sprinkle dosage form development. An attempt to propose a suitable gel vehicle was also undertaken.

## MATERIALS AND METHODS

### Materials

Diazepam was kindly donated by Polfa Tarchomin (Warsaw, Poland). The pellets were composed of microcrystalline cellulose (Vivapur PH101, JRS Pharma, Rosenberg, Germany) and lactose monohydrate (Sorbolac 400, Meggle, Wasserburg, Germany). The minitablets additionally contained croscarmellose sodium (Ac-di-sol, FMC BioPolymer, Newark, USA) as a superdisintegrant, sodium stearyl fumarate (Pruv, JRS Pharma, Rosenberg, Germany) as a lubricant and hypromellose (Pharmacoat 606, Shin-Etsu Chemical, Tokyo, Japan) as a binder. Carmellose (CMC)—high viscosity carboxymethylcellulose sodium (Sigma-Aldrich, Steinheim, Germany; viscosity of a 2.0% aqueous solution 5000 mPas) and carbomer (PAA)—and Carbopol 974P NF (Lubrizol, Brussels, Belgium; viscosity of a 0.5% aqueous solution 8000 mPas) were used to prepare gel vehicles for pellets and minitablets. Applesauce (Gerber, Nestle, Poland) was used as a soft food vehicle. Milk with 3.2% fat (Laciate, Mlekpol, Poland) and apple juice (Tymbark, Maspex, Poland) were used as liquid carriers.

### Methods

#### Production of Pellets

Pellets with diazepam were produced using an extrusion and spheronization technique. In the first step, diazepam (0.5% w/w) was mixed with diluents, microcrystalline cellulose (19.75% w/w) and lactose monohydrate (79.75% w/w), and the dry mixture was wet with water (40% w/w). The wet mass was extruded using a Caleva Extruder 25 (Caleva, Dorset, UK), and the extrudate was spheronized in a Caleva 120 spheronizer (Caleva, Dorset, UK) for 8 min with rotation speed of 1500 rpm. These parameters were chosen in a preliminary study to ensure the best sphericity and size uniformity of pellets. The wet pellets were dried at 50°C and screened to collect units with sizes of 1–1.25 mm for further studies.

The pellets’ sphericity was determined by calculating the ratio of orthogonal dimensions of pellets from stereomicroscopic pictures (Opta-Tech, Warsaw, Poland). From the captured image for 10 pellets, the longest axis was determined as the length (l), and the line perpendicular to the midpoint of the longest axis was the width (w). The ratio w/l was calculated. The ideal sphericity was a result of 1.0.

#### Production of Minitablets

The minitablets contained 0.5% (w/w) of diazepam and the following excipients: microcrystalline cellulose (56.1% w/w), lactose monohydrate (37.4% w/w) and croscarmellose sodium (2.0% w/w). The mixture was granulated with a 2% (w/w) solution of hypromellose using a high-shear wet granulation method. After granulation and drying at 50°C, a lubricant (sodium stearyl fumarate; 3% w/w) was added. The biconvex minitablets with a diameter of 2.5 mm and weight of 12 mg were prepared at a compression pressure of 250 MPa (1.2 kN) using a rotary tablet press (RTP-D8, Erweka, Germany) equipped with single punches.

#### Mechanical Strength of Pellets and Minitablets

The friability test was performed in an oscillating apparatus Model EGF-1 (Electrolab, Mumbai, India). Samples of 10 g were tested for 2 min with 140 oscillations/min and afterwards were screened using a 1.0-mm sieve. Friability was calculated as the percent of the sample weight loss.

The crushing strength test was performed using the texture analyser TA.XT plus (Stable Micro Systems, Surrey, UK). The force needed to crush a single pellet or minitablet was measured using a stainless cylinder probe. The results for 20 units are presented as a mean value and standard deviation.

#### Disintegration of Pellets and Minitablets

**Fig. 1:**
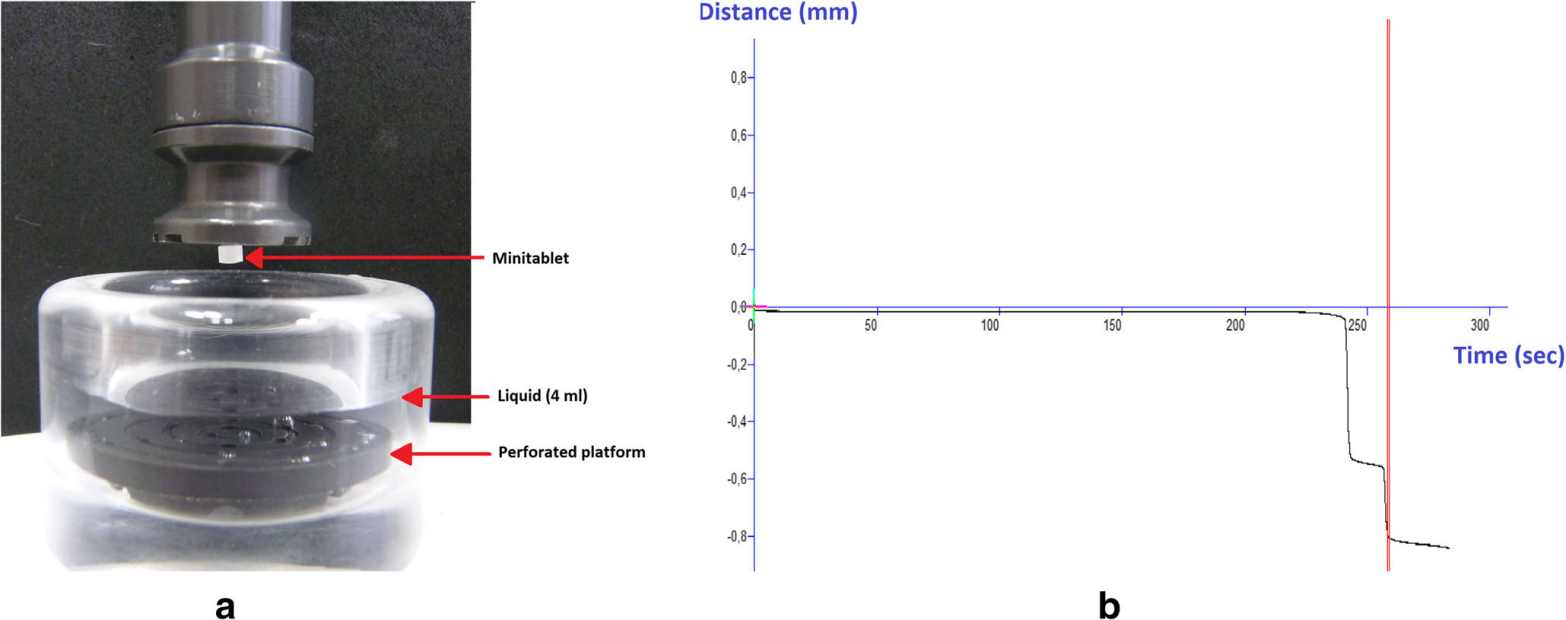
Overview of disintegration measurement using texture analyser disintegration rig: **a** the probe with attached minitablet before the test and **b** typical displacement time profile

The disintegration time was determined using a texture analyser equipped with a disintegration rig (Stable Micro Systems, Surrey, UK). A single pellet or minitablet (*n* = 10) was attached to the probe using a double-sided tape, and 4 ml of a liquid (water, milk and apple juice) was placed in a vessel with a specially perforated platform inside (Fig. 1a). The probe was brought down towards the immersed platform, and a constant 0.1 N load was exerted on the sample. The test started when the probe came into contact with the tested bead and lasted until the immersed platform surface was reached. The displacement of the probe was measured, and the distance *versus* time was plotted on a graph (Fig. 1b). The time when the displacement of the probe was not further observed corresponded to the complete disintegration of the sample (22).

Disintegration in 2.0% CMC and 0.5% PAA gels was also tested. Six pellets or six minitablets were immersed in 4.5 g of a gel on a glass Petri dish (57 mm diameter). The test was performed at room temperature, and the times until the dry cores of a pellet or minitablet were no longer observed were measured (checked every 30 s with a spatula).

#### Preparation of Gel Vehicles

Oral gels were prepared with two different polymers, carmellose sodium (2.0% CMC w/w) and carbomer (0.5% PAA w/w), *via* dispersion of the polymer in water. PAA was dispersed at room temperature, and CMC required a temperature of 60°C for dispersion. To obtain the proper viscosity, the PAA gels were neutralized (to pH 7.0) with an aqueous NaOH solution. The gels were stored for at least 24 h at 4°C and were used as vehicles after equilibration to room temperature.

#### *In Vitro* Dissolution Tests

Dissolution tests were performed in a basket (100 rpm) and paddle (75 rpm) apparatus with 500 ml of 0.1 M HCl as a dissolution medium (Pharma Test, Germany). Pellets or minitablets (1 g containing 5 mg of diazepam) were placed in a basket or vessel as loose items or dispersed in 4.5 g of a vehicle: 0.5% PAA, 2.0% CMC gels or applesauce. The samples were collected after 1 min, 3 min, 5 min, 10 min and 30 min and immediately analysed. Drug content (*n* = 6) was measured using the HPLC method (LC-2030C 3D, Shimadzu, Kyoto, Japan). A mixture of acetonitrile, methanol and 1.0% (w/w) phosphate buffer (pH 3.0) in the ratio of 18:58:24 (v/v/v) was used as a mobile phase with a flow rate of 1.0 ml/min. Chromatographic separation was performed on a LiChrosphere reversed-phase C-18 column (125 × 4 mm) with a 5-μm particle size (Merck, Darmstadt, Germany). The column temperature was 25°C, and the sample volume was 20 μl. The UV detector was set at 232 nm, and the retention time of diazepam was 2.7 min.

## RESULTS AND DISCUSSION

**Fig. 2:**
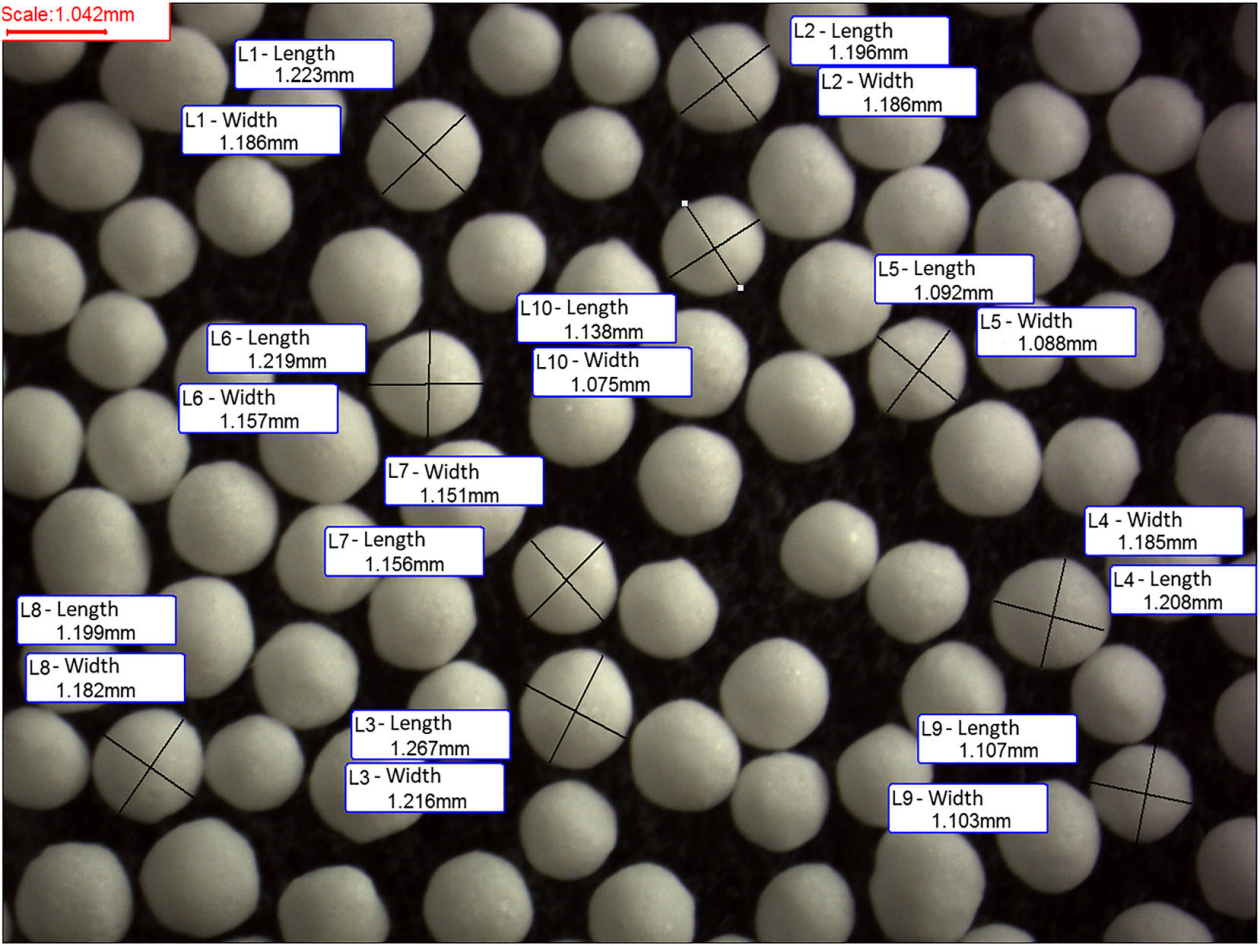
Stereoscopic microscope image of pellets with the dimensions for calculation the sphericity

The sieve analysis demonstrated that all prepared pellets were in the size range of 0.8–1.4 mm, with the main fraction (60%) in the range of 1.0–1.25-mmE, and this fraction, with good size uniformity, was selected for further studies. Moreover, good sphericity was demonstrated in microscopic pictures (Fig. 2), and high sphericity index 0.98 was calculated. The minitablets met the European Pharmacopoeia (9th edition) requirements for mass uniformity with an average mass of 12.1 mg and the maximum percentage deviation from mean value of 2.9%. The obtained dosage forms were characterized by good mechanical strength, which is required for packing into capsules or sachets. The friability was below 0.4%, and the hardness was 12.4 N (± 1.4 N) and 15.5 N (± 0.89 N) for pellets and minitablets, respectively.

In comparison with liquid formulations, solid oral dosage forms, even if uncoated, have significant potential to reduce the unpleasant taste of the active substance, on condition that they are not disintegrated or dissolved before swallowing. To assess the maintenance of the taste-masking properties after mixing with small volumes of liquid or semisolid vehicles, the disintegration test was performed. The standard pharmacopoeial disintegration test was inapplicable because of the small size of the prepared dosage forms and difficulties with the determination of the test endpoint. An alternative test with a texture analyser equipped with a disintegration rig was used. This test allowed for better detection of disintegration time. The small volume of liquid used in the test (4 ml) was also similar to the amount of vehicle used to disperse the sprinkle formulation in a spoon. The constant force applied to the sample also mimicked the tongue pressure that induces disintegration *in vivo*. This aspect should be considered, especially in patients with swallowing difficulties. Water, apple juice and milk were chosen as frequently used liquid vehicles which can facilitate the dosage form administration. They have different composition and properties, for instance pH value (16). The test was determined to show if these differences may affect the disintegration time of particles.

The tests performed in water showed that the pellets disintegrated within 3 min and minitablets disintegrated within approximately 1 min (Table I). Similar results were obtained in the tests performed in apple juice, but the disintegration time in milk was delayed to approximately 4 min for pellets and minitablets. This can be most probably related to the fat present in milk, reducing the access of water to the particle core. Larger deviations in the results were observed for pellets, which may be explained by the unstable position of the pellet under the probe due to its spherical shape, and even small changes in the measured distance resulted in large RSD (relative standard deviation) values for such small objects. However, this fast and simple test clearly showed how the type of vehicle used to disperse the sprinkles influenced the disintegration time of the investigated beads. In case of rapidly releasing formulations (within 15 min or less), the disintegration test can be used instead of dissolution testing, and the test can be more discriminating in the early development stages (23).

**Table I:** Disintegration Time of Pellets and Minitablets in Liquid Carriers as Measured Using a Texture Analyser (*n* = 10)

	Water		Milk		Apple juice	
	Mean [min/sec]	RSD [%]	Mean [min/sec]	RSD [%]	Mean [min/sec]	RSD [%]
Pellets	*2:56*	35.6	*4:27*	38.0	*3:11*	35.9
Minitablets	*0:59*	16.4	*4:07*	9.8	*1:07*	25.5

The prepared dosage forms disintegrated very fast in liquids. Therefore, in the next step, semisolid vehicles were tested as a potential aid for the administration of pellets and minitablets. CMC and PAA gels with viscosity in the range of 5000–10,000 mPas showed the best application properties (ductility and texture) for dispersing multiparticulate dosage forms (18). Additionally, good palatability may be provided because of the taste-masking properties of the gels and lubrication of the particles, which impact the mouthfeel (24). Based on these results, disintegration in 2.0% CMC and 0.5% PAA gels was tested. The gels were characterized by viscosities (measured at the shear rate 10 s^−1^) of 5000 mPas and 8000 mPas, respectively.

An attempt to use the texture analyser equipped with a disintegration rig to determine the disintegration of the units in the presence of the viscous semisolid carriers was not successful. The applied force of 0.1 N was too small to penetrate the gel surface. Modification of the maintained force up to 1 N allowed the probe to penetrate the gel, but simultaneously caused evident compression of the particles which resulted in a falsely accelerated disintegration. Therefore, the disintegration of the particles in semisolid vehicles was performed on a Petri dish. This static test simply presented how the drug product mixed with the carrier acted on a spoon. The pellets and minitablets in PAA gel and CMC gel disintegrated within 4 min and 5 min, respectively. This time is sufficient to prepare the dosage form for administration without premature disintegration. On the other hand, it was important to assess the disintegration time of particles in semisolid vehicles to assess the effect of this process on the drug release profiles.

**Fig. 3:**
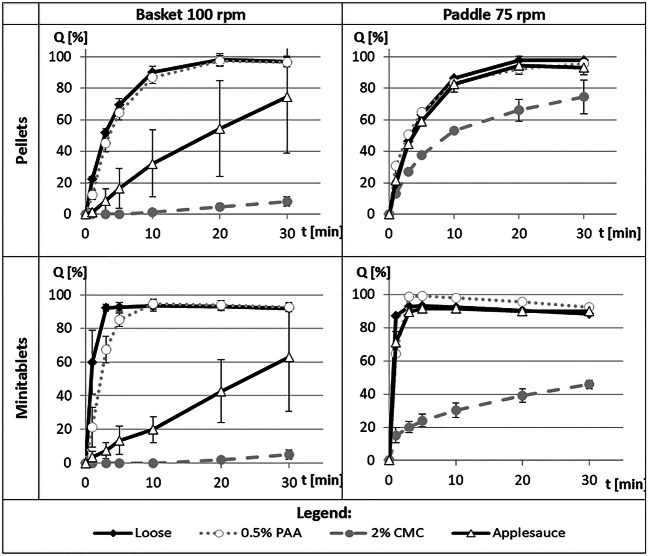
Dissolution profiles of diazepam from pellets and minitablets in a basket or paddle apparatus placed loose or dispersed in a vehicle: carbomer gel (PAA), carmellose sodium gel (CMC) or applesauce

As recommended in the recently published Guidance for Industry (13), dissolution tests were performed in a basket and paddle apparatus (Fig. 3). Hydrochloric acid (0.1 M) was chosen as the dissolution medium because the *in vivo* acidic environment of the stomach is where non-modified-release oral dosage forms must disintegrate, and the API starts to dissolve. The original loose products were tested at first. In the next steps, the pellets and minitablets were mixed with the selected vehicles on a spoon before placement in the basket or vessel. Initially, the basket apparatus was the first-choice instrument because it was easier to introduce the sample to the basket and afterwards to install it to the apparatus assembly.

Due to the presence of a disintegrating agent in the formulation (croscarmellose sodium), the loose minitablets showed an immediate dissolution of diazepam within 1–3 min, but diazepam from pellets dissolved slightly slower, with greater than 80% released within 10 min. The dissolution rate of diazepam from specific dosage forms (pellets or minitablets) was similar, regardless of the type of dissolution apparatus, paddle or basket.

In the basket apparatus, similar dissolution rates were observed for the original products and beads dispersed in a carbomer gel (0.5% PAA). In contrast, pellets and minitablets dispersed in a carmellose gel (2.0% CMC) exhibited very slow diazepam release of only 10% during 30 min. It was clearly visible that within the first 60 s of the test, the carbomer gel dissolved and the particles easily disintegrated. The carmellose gel, on the other hand, did not dissolve throughout the study and blocked the access of dissolution medium to the tested units.

Applesauce was selected for comparison as the most common soft food used for sprinkle formulations. The units dispersed in this medium showed only partial dissolution in a basket apparatus, with high deviation of the results. A basket mesh obstruction by fragments of the food vehicle was observed, and tests in a paddle apparatus were also performed.

In contrast to the basket apparatus, the beads dispersed in applesauce and tested in a paddle apparatus exhibited a fast dissolution that was comparable to the loose units and units dispersed in 0.5% PAA gel. For pellets or minitablets dispersed in CMC gel, the dissolution was still significantly slower, even when tested in a paddle apparatus. After 30 min, less than 80% of the declared dose was released (approximately 70% for pellets and 50% for minitablets). The structure of CMC gel was preserved after this time, unlike in PAA gel, which dissolved in HCl very fast and did not influence the drug release process.

Carbomer gels are prepared *via* neutralization to pH 7.0 to achieve the maximum viscosity, when the acidic groups in PAA molecules become completely dissociated. Therefore, the acidic groups in carbomer change again into an undissociated form during the dissolution tests in 0.1 M hydrochloric acid, and the gel structure becomes less viscous, which results in the release of API without slowing (25).

Sprinkling the pellets and minitablets with diazepam on a carbomer gel was similar to mixing with a semisolid food, which was demonstrated for the applesauce. However, the use of this standard gel vehicle appeared safer because the composition is simple and reproducible, which allows prediction and avoidance of potential interactions of the active substance with the components of the carrier (26). It is also very likely that the carbomer gel will not affect the drug release *in vivo* because the same mechanism of viscosity drop in acidic pH occurs in the stomach.

## CONCLUSIONS

The present study showed how the type of a dispersing vehicle influenced the *in vitro* properties of a dosage form designed for sprinkling. An alternative method for the determination of the disintegration of pellets and minitablets in liquids, which better correlated with conditions in the mouth compared with a pharmacopoeial test, was introduced. The limitation of this method is the applicability only to low viscosity carriers because problems with the endpoint determination occurred for high viscosity gels. The standard dissolution tests performed for pellets and minitablets dispersed in semisolid vehicles demonstrated the validity of these tests during the development of sprinkle formulation. The results showed that the carbomer gel may be an excellent vehicle for the sprinkles due to the pH-dependent viscosity because the stiff gel structure was not preserved in an acidic pH (stomach), and the viscosity of the vehicle did not influence the release of diazepam from pellets and minitablets. Furthermore, the use of a paddle apparatus may be recommended for testing these formulations rather than a basket apparatus because more reliable and reproducible results were obtained.
